# Predicting treat-and-extend outcomes and treatment intervals in neovascular age-related macular degeneration from retinal optical coherence tomography using artificial intelligence

**DOI:** 10.3389/fmed.2022.958469

**Published:** 2022-08-09

**Authors:** Hrvoje Bogunović, Virginia Mares, Gregor S. Reiter, Ursula Schmidt-Erfurth

**Affiliations:** ^1^Laboratory for Ophthalmic Image Analysis, Department of Ophthalmology, Medical University of Vienna, Vienna, Austria; ^2^Department of Ophthalmology, Federal University of Minas Gerais, Belo Horizonte, Brazil

**Keywords:** neovascular age related macular degeneration, optical coherence tomography, anti-VEGF (vascular endothelial growth factor), image analysis, retina, machine learning, AI

## Abstract

**Purpose:**

To predict visual outcomes and treatment needs in a treat & extend (T&E) regimen in neovascular age-related macular degeneration (nAMD) using a machine learning model based on quantitative optical coherence tomography (OCT) imaging biomarkers.

**Materials and methods:**

Study eyes of 270 treatment-naïve subjects, randomized to receiving ranibizumab therapy in the T&E arm of a randomized clinical trial were considered. OCT volume scans were processed at baseline and at the first follow-up visit 4 weeks later. Automated image segmentation was performed, where intraretinal (IRF), subretinal (SRF) fluid, pigment epithelial detachment (PED), hyperreflective foci, and the photoreceptor layer were delineated using a convolutional neural network (CNN). A set of respective quantitative imaging biomarkers were computed across an Early Treatment Diabetic Retinopathy Study (ETDRS) grid to describe the retinal pathomorphology spatially and its change after the first injection. Lastly, using the computed set of OCT features and available clinical and demographic information, predictive models of outcomes and retreatment intervals were built using machine learning and their performance evaluated with a 10-fold cross-validation.

**Results:**

Data of 228 evaluable patients were included, as some had missing scans or were lost to follow-up. Of those patients, 55% reached and maintained long (8, 10, 12 weeks) and another 45% stayed at short (4, 6 weeks) treatment intervals. This provides further evidence for a high disease activity in a major proportion of patients. The model predicted the extendable treatment interval group with an AUROC of 0.71, and the visual outcome with an AUROC of up to 0.87 when utilizing both, clinical and imaging features. The volume of SRF and the volume of IRF, remaining at the first follow-up visit, were found to be the most important predictive markers for treatment intervals and visual outcomes, respectively, supporting the important role of quantitative fluid parameters on OCT.

**Conclusion:**

The proposed Artificial intelligence (AI) methodology was able to predict visual outcomes and retreatment intervals of a T&E regimen from a single injection. The result of this study is an urgently needed step toward AI-supported management of patients with active and progressive nAMD.

## Introduction

Age-related macular degeneration (AMD) is a complex, multifactorial and heterogeneous disease with its late-stage neovascular AMD (nAMD) form leading to a rapid and severe vision loss (1). Anti-vascular endothelial growth factor (anti-VEGF) therapy has revolutionized the treatment of nAMD (2). Anti-VEGF drugs are highly effective in drying the retina, but have a few important shortcomings. The high drug costs and the need for frequent injections are placing a large socioeconomic burden on healthcare systems as well as patients. In particular, the need to comply with frequent hospital visits and injections creates difficulties for patients to meet such an intensive schedule, especially in developing countries where tertiary centers are concentrated in few reference cities, but also in locations with adequate infrastructure. As a result, the real-world outcomes are largely inferior to the ones observed in the clinical trials (3, 4). Lastly, inter-individual treatment requirements are highly heterogeneous and there is a clear need to tune the anti-VEGF treatment to an individual’s disease profile, (5) to reduce the number of visits and injections, while still improving patients’ visual function. However, tools and imaging biomarkers to predict these individual requirements are largely unknown and remain an unmet medical and socioeconomic need in developed and in developing countries (6).

Currently, there is no universally accepted treatment regimen that balances the frequency of treatment needed to achieve the optimal visual outcomes with the burden of long-term, frequent and high-cost treatment (7). *Pro re nata (PRN)* regimen aim at decreasing the injection load by injecting only “when needed,” but require monthly visits. On the other hand, *treat-and-extend (T&E)* was designed as a proactive treatment that can decrease the number of visits, while maintaining a fluid-free macula with proactive intervention before fluid recurs (8–10). T&E has become the most frequently used treatment regimen as in contrast to PRN, it can enable patients to go as long as 12 weeks between office visits and injections without monitoring visits (11, 12). In addition to lessening the burden on patients, T&E regimen help clinicians to cope with the complexity and unpredictability of nAMD individual response to therapy. Yet, the price to pay are more injections and a somewhat unpredictable path to identify the right interval and no clear control of disease activity over long-term maintenance.

Optical coherence tomography (OCT) is the standard of care and the most commonly used imaging modality in ophthalmology, providing real-time information on retinal structure and assessing response to therapy (13). Clinicians came to embrace the use of OCT imaging as the basis for dosing with anti-VEGF drugs (14). Its fast scanning with microscopic resolution creates large and detailed 3D volumetric scans of the retina. However, the big data format of OCT images has started to dramatically outperform the capacity of a human expert to adequately evaluate the diagnostic and predictive information contained within, and the discrepancy between the imaged details and clinical conclusive insight is growing rapidly (6).

Artificial intelligence (AI) has a large potential in enabling high performance medicine in general (15) and automating retinal image analysis in particular (16). Driven by deep learning, (17) AI has recently enabled fully autonomous differential diagnosis from OCT scans, (18, 19) all operating at the level of a retinal specialist. In the context of anti-VEGF treatment support, there is still a largely underexplored potential of AI to help guide long-term management and further personalize it (20). In this paper, we aim at building an AI-based model capable of predicting for each patient with nAMD the visual response to anti-VEGF injections as well as the treatment requirements during a T&E regimen. Our core hypothesis is that the predictive signs of these future outcomes and needs are contained in the OCT scans of the retina acquired at the very initial phase of the treatment course. Using automated retinal image analysis based on deep learning, a set of quantitative spatio-temporal biomarkers were extracted from a pair of consecutive OCT scans only 4 weeks apart, characterizing the retinal condition and its response to the first anti-VEGF injection. Machine learning was then applied to train a predictive model of the future visual outcomes and treatment needs. Our model was trained and evaluated on the 1-year data of the T&E treatment arm of a prospective and standardized clinical trial involving treatment-naïve nAMD patients.

## Materials and methods

### Participants and imaging protocols

This *post-hoc* analysis was performed on the OCT scans, clinical and demographic data of eyes of patients undergoing a T&E regimen within the TReat and extEND (TREND) clinical trial (ClinicalTrials.gov identifier: NCT01948830). TREND was a 12-month, phase IIIb, randomized, visual acuity assessor-masked, multi-center, interventional study assessing the efficacy and safety of T&E vs. monthly 0.5 mg ranibizumab intravitreal injections in patients with newly diagnosed nAMD (21). In the TREND T&E arm, eyes were initially treated at monthly intervals until disease activity resolved. When fluid was not present anymore, the interval was extended by 2 weeks to a maximum of 12-weeks. When fluid was again present, the interval was shortened by 2 weeks up to a minimum of 4 weeks. The opportunity of extending the interval was limited to two attempts. At every visit, best corrected visual acuity (BCVA) was measured, and an OCT acquired. Disease activity was assessed by visual acuity and OCT criteria according to the investigator’s judgment, based on the presence of intraretinal fluid (IRF) or subretinal fluid (SRF) (21).

The OCT scans were macula-centered covering the volume of 6 mm × 6 mm × 2 mm and were acquired with a Cirrus HDOCT III (Carl Zeiss Meditec, Inc., Dublin, CA, United States) having 128 B-scans with 512 × 1024 pixels, Spectralis (Heidelberg Engineering, Heidelberg, Germany) having 49 B-scans with 768 × 496 pixels, or Topcon OCT-2000 (Topcon, Tokyo, Japan) having 128 B-scans with 512 × 885 pixels. The majority of the scans were acquired with Spectralis (65%), followed-by Cirrus (25%), and Topcon (10%). The OCT scans of all patients were collected centrally by Vienna Reading Center (VRC) according to a predefined imaging protocol. OCT images of patients that gave informed consent for research analysis were transferred *post-hoc* to the Laboratory for Ophthalmic Image Analysis (OPTIMA) at the Medical University of Vienna in a pseudonymized format to perform the AI-based analysis. The *post-hoc* analysis presented here was conducted in compliance with the Declaration of Helsinki and approval was obtained by the Ethics Committee at the Medical University of Vienna (EK Nr: 1246/2016).

### Automated optical coherence tomography analysis

**FIGURE 1:**
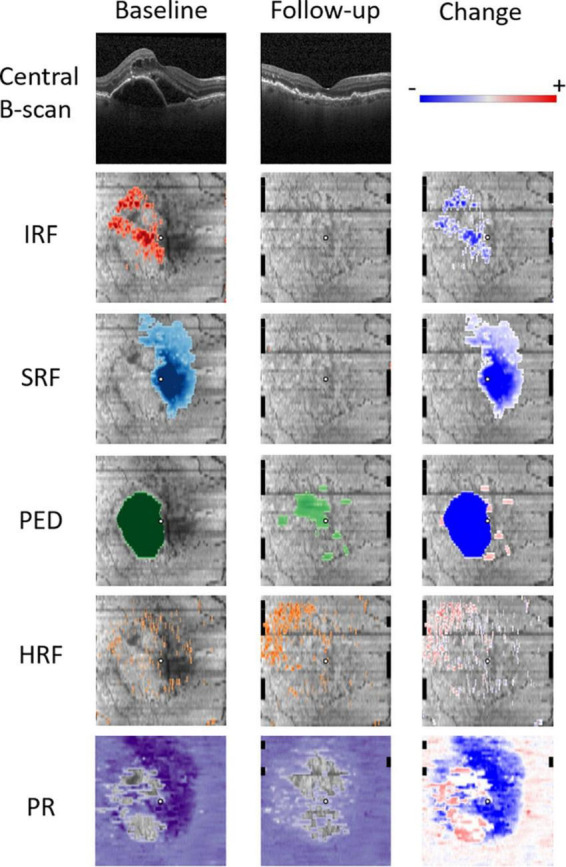
Quantitative OCT biomarker thickness maps: intraretinal fluid (IRF) in red, subretinal fluid (SRF) in blue, pigment epithelium detachment (PED) in green, hyperreflective foci (HRF) in orange, and photoreceptor (PR) layer thickness in purple. Follow-up scan was acquired 4 weeks after the initial treatment. Change refers to the difference between the baseline and the first follow-up.

To semantically describe the content of the volumetric OCT scans, a series of fully automated image segmentations was performed to detect and quantify the following OCT imaging biomarkers known to be associated with the nAMD progression (Figure 1).

#### Retinal fluid compartments

Detection and quantification of fluid was performed on every B-scan of an OCT volume with an evaluated deep-learning based image segmentation method (22). Every pixel was classified with a multi-scale convolutional neural network (CNN), and assigned a probability of belonging to one of the four classes: background, retina, IRF or SRF. In addition, pigment epithelial detachment (PED) was identified as a region in-between RPE and Bruch’s membrane, which were automatically segmented using the Iowa Reference Algorithms (23, 24).

#### Hyperreflective foci

We defined HRF as small, dot-shaped lesions with equal or higher reflectivity than the retinal pigment epithelium (RPE). Analogous to fluid quantification, HRF were segmented with a CNN model previously developed for this purpose, which has been shown to perform similar to a trained human image grader (25). Every pixel of the scan was assigned a probability of belonging to an HRF.

#### Photoreceptors

Photoreceptor layer segmentation was performed with a previously developed and validated CNN (26). The method delineates two surfaces defined as the inner boundary of the IS/OS junction and the outer boundary of the outer photoreceptor segments. The photoreceptor integrity was then represented in the form of a 2D thickness map defined by the distance between the two segmented surfaces.

### Predictive model development

The 2D en-face thickness maps of the photoreceptors, HRF, as well as the IRF, SRF, and PED as quantitative biomarkers were computed from the segmented OCT scans at the baseline and the first follow-up visit, which we used as an *observation period* (Figure 1). The thickness maps obtained from the scans acquired with Spectralis were resampled with bilinear interpolation to match the 128 B-scan resolution of the ones acquired with Cirrus/Topcon. To measure the *change* of retinal morphology after the first injection, the difference maps between the follow-up and the baseline visits were computed. To decrease the dimensionality of our representation, we spatially divided the retina into three regions corresponding to the central 1 mm, a parafoveal ring (1 to 3 mm) and a perifoveal ring (3 to 6 mm). Then, for each imaging biomarker a mean value of each of the three spatial regions was computed to transform a 2D thickness map into a 3-dimensional feature vector. The features from biomarkers IRF, SRF, PED, and HRF were represented as volumes in nanoliters (nl), and PR as thickness in μm. The five biomarkers over three time points and intervals (baseline, follow-up, change) form a set of 45 (5 × 3 × 3) quantitative features characterizing the retinal pathomorphology in a spatio-temporal manner. To this set of OCT-derived features we further included the BCVA values at baseline, the follow-up visits and the change, as well as two available demographic features: age and sex. In total, each individual eye was hence characterized with a 50-dimensional feature vector, which served as a set of predictors for training predictive models of treatment intervals and visual responders.

#### Treatment-interval by patient groups

Two patient groups were defined based on the treatment interval established during the trial. The first group corresponded to *non-extendable* patients, the ones that primarily stayed at or later fell-back to 4–6-week maximum intervals. The second group, corresponded to the *extendable* patients, the ones that reached and maintained a treatment interval of at least 8-weeks or more. For the interval to be considered as maintained a patient had to have received at least two treatments with such an extended interval.

#### Treatment-responder by patient groups

To identify groups of patients with respect to their visual response to treatment, BCVA trajectories were modeled with latent class mixed models (LCMM) using *lcmm* package for R (R Foundation for Statistical Computing, Vienna, Austria) (27). The linear mixed model has become a standard statistical tool to analyze longitudinal measurements and LCMM extends it to account for non-observed heterogeneity that may exist in the population, in our case this being the responder/non-responder status. BCVA trajectories produced by the latent process were modeled by a quadratic function of time, and the model included no baseline covariates. We assumed a large number of latent classes (six) to identify diverse patient subgroups in an unbiased way based on their visual response alone, expecting to later merge them into the two clinically relevant groups indicating responder/non-responder.

#### Machine learning

To build a predictive model of the treatment interval groups, and the visual responder groups, a random forest classifier (28) was trained on the spatio-temporal feature vectors. Random forest was grown with 2,000 trees, minimum node size of 1, and 7 features randomly sampled as candidates at each split of a tree. For this, R-Package “randomForest” (v4.6-12) that implements Breiman and Cutler’s random forests for classification and regression was used, on a hardware with a 3.30 GHz CPU and 32GB of RAM. The performance of the predictive model was evaluated using a stratified 10-fold cross-validation. Thereby, the cohort is stratified into 10 equal sized subsamples with the condition that the proportion of each class in all the folds is approximately equal, ensuring that each fold is representative of the entire cohort. At each of the 10-folds, 90% of the data was used for training and validation, and 10% for test. Out-of-bag (OOB) error on the training set was used as a validation error to tune the hyperparameters of the random forest.

### Statistical analysis

The predictive model produces a probabilistic estimate of a sample belonging to each class, and the performance was measured with an area under the receiver operating characteristic (ROC) curve (AUC) and summarized by the *sensitivity* and *specificity* at an optimal operating point. Confidence intervals (95%) of AUC were obtained with 1000 bootstraps. To evaluate the predictive role of the individual features, the *importance measure* we used relied on permuting the value of a feature and measuring how much the permutation decreases the prediction accuracy of the model.

## Results

### Study cohort and descriptive analysis

Out of 650 participants in TREND, 544 gave an informed consent for research use of the data. From that cohort, the 270 patients that were randomized to the T&E arm were considered for the purpose of this study. For predictive modeling, a total of 10 patients were discarded due to a missing OCT scan from the first two visits (baseline or the first follow-up). Furthermore, 22 eyes were lost to follow-up, 5 were found to have bad quality scans, and 5 were found to have two or more visits missing injections or to have more than 12-week intervals, deviating from the trial protocol. Finally, 228 evaluable eyes from as many patients were considered for cross-validation. The mean (SD) age of these patients was 75.2 (± 8.2) years (range 51–90); 55% were female. Mean (SD) baseline BCVA was 58.4 (± 13.2) letters and mean total number of injections received over 12 months was 9.1.

**FIGURE 2:**
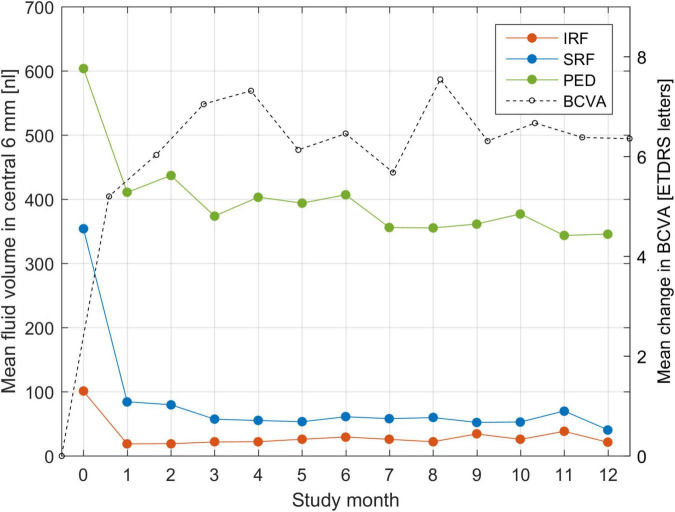
Mean fluid volume in the central 6-mm during the course of T&E treatment for each of the three fluid types: intraretinal (IRF), subretinal (SRF) and sub-RPE (PED). Fluid volumes are expressed in nanoliters (nl) and put in correspondence with the mean change in BCVA from baseline.

An analysis of population mean fluid volumes and BCVA trajectory during the trial is shown in Figure 2. One can observe a rapid decrease in IRF volume already after a single injection. Similarly, SRF decreases quickly, but slower than IRF and it requires more injections for complete resolution. PED decreases in volume after the first injection, but never diminishes further and remains of substantial size throughout the treatment course. The largest increase in mean BCVA was consistently noted after the very first injection. This confirms most strikingly the general effectiveness and the utility of anti-VEGF drugs in clearing the retina from fluid and improving the visual function.

**FIGURE 3:**
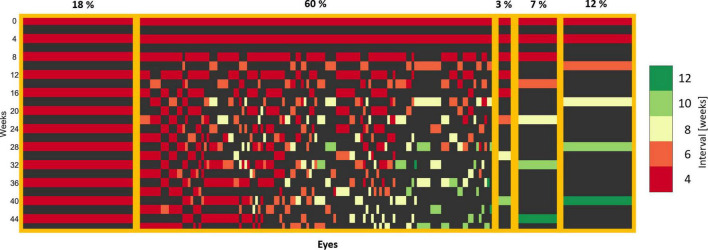
Treatment patterns of eyes in the T&E arm. Each column corresponds to one eye. Each row corresponds to a visit and the corresponding 4-week interval. A loading dose of two injections is apparent, as all the eyes received an injection at the baseline and the first follow-up visit. The eyes with an individual treatment were sorted by the last extension interval.

Patients grouped by the frequency pattern of the received treatment are displayed in Figure 3. We observed the following patterns: All patients effectively received a loading dose consisting of two consecutive injections at the baseline and the first follow-up. Further, 18% of patients required intensive monthly treatment (left side of Figure 3) and 22% were continually extended as soon as a dry retina was achieved after 2–4 initial injections (right side of Figure 3). The remaining 60% of the patients experiencing an individualized treatment course due to a variable response pattern. Overall, 55% of patients were extendable per our definition, reaching and maintaining long (8, 10, 12 weeks), while the other 45% were non-extendable, staying at short (4, 6 weeks) treatment intervals.

**FIGURE 4:**
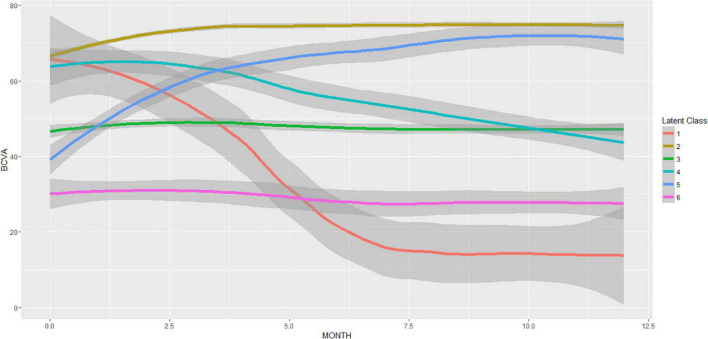
Latent class mixed model (LCMM) with six latent classes of the BCVA trajectories during the T&E treatment. The resulting mean (± 95% CI) BCVA trajectories are displayed. The number of patients per latent class was 1:4, 2:173, 3:58, 4:12, 5:15, 6:8. Responders (latent classes: 2,5) and non-responders (latent classes: 1,3,4,6) are clearly distinguishable.

Identification of responders/non-responders following the LCMM modeling with six latent groups is shown in Figure 4. The clustering of the BCVA time trajectories revealed responders and non-responders for two different baseline BCVA levels: High (BCVA > 50 Letters), and Low (BCVA < 50 Letters), with their respective prevalence provided in Table 1.

**TABLE 1:** Visual function responder/non-responder subgroups stratified by baseline (BSL) visual acuity. The number of patients and their prevalence is reported for each subgroup.

Baseline BCVA (Letters)	Responder	Non-responder
High (≥50)	173 (64.1%)	16 (05.9%)
Low (<50)	15 (05.6%)	66 (24.4%)
	69.7%	30.3%

### Artificial intelligence for predicting best corrected visual acuity treatment responders/non-responders

**FIGURE 5:**
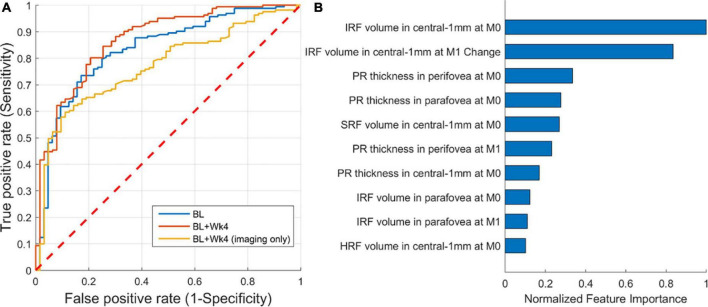
Performance of predicting responders/non-responders. **(A)** Receiver operating characteristic (ROC) of prediction from: baseline (AUC = 0.83), baseline + follow-up (week 4) (AUC = 0.87), and baseline + follow-up restricted to imaging features only (AUC = 0.77). **(B)** The 10 most important imaging-only features from the first two visits [baseline (M0) and month one (M1)] for predicting responders/non-responders.

A ROC curve of the predictive model, representing the trade-off between specificity and sensitivity, is shown in Figure 5A. The AUC for predicting the responders was 0.87 (CI: 0.80–0.91). The AUC was 0.83 and 0.77 when considering only baseline features or only the imaging baseline + follow-up features, respectively. An operating point that maximizes both sensitivity and specificity would yield a sensitivity and specificity of 80%. The drop in predictive performance of 0.1 AUC when excluding non-imaging BCVA features clearly indicates that BCVA at the latest observed time-point was the single most important prognostic factor. Sub-analysis of the performance of the model based on imaging features only, revealed a performance of 0.72 (CI: 0.55–0.86) and 0.70 (CI: 0.58–0.81), for Low and High baseline BCVA subgroups, respectively.

A detailed analysis of feature importance for predicting responders confirmed that BCVA at week 4 and BCVA at baseline were the two most predictive factors. Focusing on imaging features only (Figure 5B), we found that the volume of IRF and its change from baseline to follow-up were the two most predictive imaging features relevant for BCVA outcomes. The two demographic variables were not found to play a role.

### Artificial intelligence for predicting treatment requirements

**FIGURE 6:**
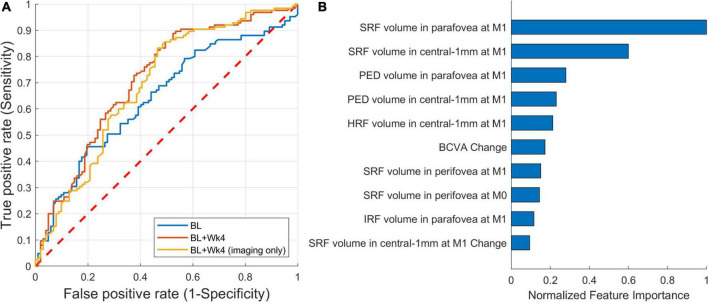
Performance of predicting extendable patients. **(A)** Receiver operating characteristic (ROC) of prediction from: baseline (AUC = 0.64), baseline + follow-up (week 4) (AUC = 0.71), and baseline + follow-up restricted to imaging features only (AUC = 0.69). **(B)** The 10 most important imaging features from the first two visits [baseline (M0) and month one (M1)].

A ROC curve of the predictive model is shown in Figure 6A. The AUC for predicting the extendable from the non-extendable group was 0.71 (CI: 0.64–0.78). The AUC was 0.64 and 0.69 when considering only the baseline features or only the imaging baseline + follow-up features, respectively. For predicting the actual treatment intervals, the false positives (patients wrongly predicted to be extendable) are considered more adverse than the false negatives (patients wrongly predicted to remain at short treatment intervals). Thus, the operating point should be set to favor specificity over sensitivity. Using such conservative operating points at 80% specificity, the predictive model detected the extendable patient groups with a sensitivity of 46% (Figure 6A). The drop in predictive performance from 0.71 to 0.64 when only the baseline scans were considered shows the importance of observing the retinal response after an injection is given. When excluding non-imaging BCVA features the AUC remained similar, hence for this task BCVA was not a very important prognostic factor.

The top 10 most Important features for this predictive task are shown in Figure 6B. The feature importance was correlated with the response to the initial anti-VEGF treatment, with the most important features being measured after the first injection. The list was led by features related to SRF, unlike the previous model where IRF-related features were prominent. In comparison to the imaging data, only the BCVA change from baseline to the first follow-up was being featured within the top 5% of the most important features. Again, the two demographic features were not found to have a predictive role.

## Discussion

Our study shows that artificial intelligence is able to classify patients in responders or non-responders, and predict the individual treatment needs, based on visual acuity and imaging biomarkers solely from the first two OCT exams (baseline and 1 month after the injection). The AUC for identifying responders varied between 0.77 and 0.87 depending on which information was made available to the model. Though baseline BCVA was found to be the most important predictor, the imaging-based biomarkers, in addition to being an objective measure, showed their value in distinguishing outcomes of patients with similar baseline BCVA. The predictive model of future treatment intervals reached an AUC of 0.71 representing the variability of predicting future treatment needs at the individual level. The “fair” performance illustrates the difficulty of this prediction task, which is partly hindered by the short 1-year duration of the trial, not allowing to identify the stable retreatment interval for every patient.

Treat-and-extend has become the most popular treatment regimen (11, 12) due to a decrease in the number of visits with non-inferior visual outcomes compared to monthly injections in clinical trials (21). However, the discussion regarding the most appropriate treatment regimen is still ongoing because T&E may imply overtreatment when a patient could be extendable for more than 2 weeks at a time and by its proactive nature of intervention. Therefore, the use of AI for individual treatment prediction has a high potential to avoid unnecessary visits as well as procedures which potentially lead to severe complications such as endophthalmitis or retinal detachment. The TREND study nicely represents the heterogeneity in anti-VEGF treatment response even in a clinical trial cohort, where inclusion and exclusion criteria make the patient sample rather standardized. On one hand, around 18% of patients required intensive monthly treatment (non-extendable). On the other hand, 22% were continually extended after a dry retina was achieved after 2–4 initial injections (extendable), the maximal interval in TREND. The remaining 60% of the patients experienced an individual treatment pattern with changing fluid status of the macula. There is therefore a clear need to tune the anti-VEGF treatment in nAMD to a personalized regimen. In the absence of clear inclusion/exclusion criteria and an overwhelming number of patients in the real-world, this need is even more urgent.

Moreover, our analysis impressively demonstrates the overall high levels of exudative activity in nAMD disease: Half of the patients cannot be extended to 2 month intervals or beyond which is distinctly above the retreatment regimens in clinical practice globally (29). Obviously, the TREND study administered ranibizumab which used to be the most frequently applied substance in anti-VEGF therapy. In 2022, aflibercept is the blockbuster drug in terms of revenue, however, little difference was found in respect to efficacy and durability between both drugs when patients were either switched (30) or compared head-to-head in a prospective trial (31). The RIVAL study looked at a treat-and-extend regimen and found that neither aflibercept nor ranibizumab were superior to the other regarding 12-month average visual acuity gains and injection numbers. The search for novel substances with longer *in vivo* durability is therefore a most busy field with brolucizumab leading to severe complications (32) and faricimab with a combined anti-VEGF and anti-angiopoetin profile (33). Faricimab was found to be equivalent in BCVA outcomes, but a similar need for frequent retreatments in the group with high disease activity. Long-term maintenance can be achieved using intraocular refillable implants (34). At month 9, the mean CFT change from baseline was similar in the Port Delivery System 100-mg/ml and monthly intravitreal ranibizumab 0.5-mg arms which makes the device a promising alternative for eyes with high leakage activity. AI tools may reliably identify those candidates already following the first injection. Globally, bevacizumab is the most frequently used compound due to its low cost, and trials have already demonstrated differences in fluid resolution speed and durability (35). Such benchmarking of anti-fluid capacities of substances can be easily performed by comparing AI-based fluid resolution patterns, analogous to the one shown in Figure 2. The arrival of biosimilars will substantially change the landscape of agents used in nAMD in respect to healthcare budgets (36). For optimization of regimens and outcomes, however, efficacy profiles will be needed to start adequate long-term care from the therapeutic start.

The introduction of OCT imaging allowed first qualitative, and later also quantitative assessments of pathomorphologic features of the retina, becoming essential in active monitoring, treatment decisions and patient visit management on an individualized basis. Yet, as imaging technology becomes more sophisticated, the discrepancy between image details and clinical interpretation is growing (6). There, AI becomes a useful tool as it has been applied to retinal OCT imaging to quantify fluid, (22, 37) provide prognosis, (38, 39) and predict treatment requirements in nAMD patients undergoing an anti-VEGF PRN regimen, (40, 41) and more recently treatment demands in real-world cohorts (42, 43).

In this study, automated OCT quantification was performed for the following OCT imaging biomarkers associated with nAMD disease progression: macular fluid compartments (IRF, SRF, PED), which are well known to be the most important imaging biomarkers for anti-VEGF guidance, HRF that have been reported to be a negative prognostic factor for visual function in nAMD, (44, 45) and photoreceptor integrity as visualized by OCT, which are hypothesized to be important surrogate markers of treatment outcomes (46). Analyzing fluid, we observed a rapid decrease in IRF and SRF volume already after a single injection, however, SRF decreased slower than IRF, which is consistent to the literature (47). The TREND study protocol considered the presence of intraretinal or subretinal fluid as disease activity, to be treated in all cases. SRF-related features were more important features for predicting treatment requirement, unlike the previous experiment for identifying responders/non-responders, which IRF-related features were dominating. Yet, one must consider that such a retrospective analysis does not identify the optimal retreatment scenario, but what the investigators decided based on the protocol of the trial. As SRF resolves more slowly under therapy, non-extended or shortened intervals would mostly be triggered by the SRF fluid type. This does not necessarily mean that even small amounts of SRF, should be treated to achieve more visual gain. PED decreases after the first injection, but remains of substantial size throughout the treatment course even with further improvement of IRF, SRF, and BCVA, endorsing the slow response of these lesion types to anti-VEGF injections treatment (48). A treatment-agnostic large scale analysis of BCVA changes dependent on fluid volumes of all types in the HAWK & HARRIER study clearly highlighted the fact that an increase in volume in all compartments was independently associated with visual loss (49).

The closer relation of IRF compared to SRF for predicting responder/non-responders for visual outcomes, despite slower SRF depletion, can be explained by the predominant location of SRF outside of the 1 mm center macula and the high impact of foveal IRF on BCVA. In addition, the FLUID study has postulated a possible tolerance of SRF in the foveal center without having a substantial negative impact on visual outcomes (50). However recent reports showed a negative impact of SRF fluctuations on photoreceptor integrity, a positive correlation between EZ integrity on OCT images and BCVA and a correlation between residual SRF and short term-BCVA loss using AI tools (51, 52). These findings reflect that a more detailed analyses of retinal fluids and their locations are essential to predict and manage nAMD treatment. Furthermore, other biomarkers as subretinal hyperreflective material (SHRM), as well as the development of fibrosis and atrophy under the influence of different fluid types have sparked the interest of the scientific community and are associated with long term loss of visual function. Since TREND was a 1-year study, these biomarkers probably did not affect the outcomes for the first year but should be considered for any longer investigation in patients with AMD. In addition, the TREND evaluable subcohort in this study included 228 patients and due to this relatively small sample size, deep learning approaches based on CNN that would perform the prediction from the pair of raw 3D OCT scans or from the intermediate 2D feature maps were not attempted but would be a promising alternative in a similar scenario involving a larger number of participants.

There are two main distinguishing features of this work. First, we now included HRF volume and photoreceptor layer thickness, reflecting atrophy or intraretinal edema, as additional biomarkers, which have not been explored before for these predictive purposes. Second, we defined the visual outcome subgroups based on the complete patient BCVA trajectory, as opposed to relying on a visual test from a single visit at the end of the study and a predefined response threshold. We found the LCMM-based modeling to be a promising step toward the discovery of visual response subgroups and to offer a more comprehensive evaluation of the visual response. From the methodological perspective, this study follows the approach explored in previous works (38, 41–43), where the retina was first characterized with a set of quantitative biomarkers, followed by machine learning to build the predictive model. The results of predicting the treatment requirements (AUC of 0.71) fell slightly below the performance reported there, but within the confidence interval, where in Gallardo et al. (43) they obtained an AUC of 0.79, and in Bogunovic et al. (41) an AUC of 0.77 for predicting high-demand patients. It is worth noting that in these related works, the high demand corresponded to the top tercile, and the patient cohort was larger (>300), which facilitated the learning task.

This study has inherent limitations for being a *post-hoc* analysis performed on patients from one arm of the TREND study, and for the study being of 1-year duration. First, as mentioned above, the protocol oriented to always treat the presence of retinal fluid, while both intraretinal and subretinal and fluid tolerance is still a controversial topic with a strong impact on the number of injections. Second, the images were not acquired specifically for this analysis, but for the previously performed clinical trial. The algorithms applied have been developed and tested for Spectralis and Cirrus scans but not Topcon. Qualitatively, the segmentation performance on Topcon scans, which constituted only 10% of the scans, was found to be similar to the one on Cirrus, and the two scanners produced comparable image style, and signal to noise ratio. Furthermore, due to a small sample size we resorted to cross-validation and an external validation on an independent test set is required to confirm the model’s generalizability. Finally, we believe that SHRM, fibrosis, and atrophy may be important biomarkers for predicting visual acuity outcomes in nAMD, which should be considered for future investigations with longer follow-up.

Detailed analysis of OCT images using AI is becoming a powerful tool to predict anti-VEGF treatment requirement based on the quantitative fluid response pattern. Despite a more appropriate patient counseling and increased patient’s adherence to the treatment, this prediction will also improve resource management and avoid under- or over-treatment. Considering that in many developing countries the tertiary and academic centers are concentrated in few reference cities, a more rational manage of injection bursts may avoid unnecessary dislocation for patients’ visit and treatment. Forecasting of treatment requirement by automated and objective AI-based quantification of treatment response might also be useful to identify patients who benefit from the upcoming long-acting therapy approaches. Together with the advent of biosimilars, advances in AI-based image analysis may allow a novel level of optimization of the most frequently applied intervention in the entire field of medicine. Further studies are necessary, including real-world data with larger patient populations to establish an image-guided prediction for clinical decision of treatment intervals in the management of neovascular AMD.

## Data availability statement

The datasets presented in this article are not readily available because original raw data for this research were provided by the Novartis Pharma AG. Derived data that support the findings of this study are available upon reasonable request. Requests to access the datasets should be directed to HB, hrvoje.bogunovic@meduniwien.ac.at.

## Ethics statement

The studies involving human participants were reviewed and approved by the Ethics Committee of the Medical University of Vienna Submission Nr 1246/2016. The patients/participants provided their written informed consent to participate in this study.

## Author contributions

HB, VM, GR, and US-E contributed to conception and design of the study. HB performed the image and statistical analysis. HB and VM wrote the first draft of the manuscript. GR and US-E wrote sections of the manuscript. All authors contributed to manuscript revision, read, and approved the submitted version.
